# Molecular Dynamics and Self-Assembly in Double Hydrophilic Block and Random Copolymers

**DOI:** 10.1021/acs.jpcb.4c05398

**Published:** 2024-11-05

**Authors:** Achilleas Pipertzis, Angeliki Chroni, Stergios Pispas, Jan Swenson

**Affiliations:** † Department of Physics, Chalmers University of Technology, 41296 Gothenburg, Sweden; ‡ Theoretical and Physical Chemistry Institute, National Hellenic Research Foundation, 48 Vassileos Constantinou Ave., 11635 Athens, Greece

## Abstract

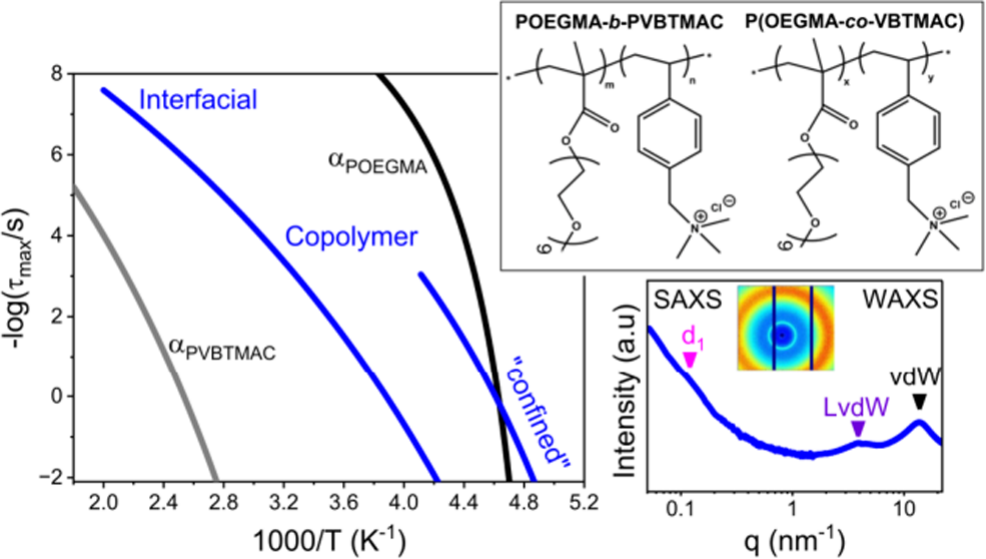

We investigate the self-assembly and dynamics of double hydrophilic block copolymers (DHBCs) composed of densely grafted poly[oligo(ethylene glycol) methacrylate] (POEGMA) and poly(vinyl benzyl trimethylammonium chloride) (PVBTMAC) parent blocks by means of calorimetry, small- and wide-angle X-ray scattering (SAXS/WAXS), and dielectric spectroscopy. A weak segregation strength is evident from X-ray measurements, implying a disordered state and reflecting the inherent miscibility between the host homopolymers. The presence of intermixed POEGMA/PVBTMAC nanodomains results in homogeneous molecular dynamics, as evidenced through isothermal dielectric and temperature-modulated DSC measurements. The intermixed process undergoes a glass transition at a temperature approximately 40 K higher than the vitrification of bulk POEGMA segments, and it shifts to an even higher temperature by increasing the content of the hard block. At temperatures below the intermixed glass transition temperature, the confined POEGMA segments between the glassy intermixed regions contribute to a segmental process featuring (i) reduced glass transition temperature (*T*_g_), (ii) reduced dielectric strength, (iii) broader distribution of relaxation times, and (iv) reduced fragility compared to the POEGMA homopolymer. We also observe two glass transition temperatures of dry PVBTMAC, which we attribute to the backbone and side chain segmental relaxation. To the best of our knowledge, this is the first time in the literature that these glass transitions of dry PVBTMAC have been reported. Finally, this study shows that excellent mixing of the two homopolymers is obtained, and this implies that different properties of this copolymer system can be tailored by adjusting the concentration of each homopolymer.

## 1 Introduction

Block copolymers are a category of polymers in which the *macromolecule* consists of two or more distinct blocks of homopolymers, through a copolymerization process.^1^ Various kinds of block copolymers have been synthesized since Szwarc first synthesized a block copolymer by using a living anionic polymerization technique.^2^ Depending on how the different monomer species are arranged in the polymer chain, copolymers can be categorized into several categories including random, alternating, block, graft, or those referred to as statistical distributions of monomers. This molecular arrangement imparts a diverse range of mechanical, thermal, and chemical properties and material classifications. Particularly, block copolymers can self-assemble into different ordered nanophases. The degree of segregation between the two blocks is dependent on the product of the interaction parameter, *χ*, and the total degree of polymerization, *N*.^1^

Double hydrophilic block copolymers (DHBCs), composed of two water-soluble host constituents of different chemical natures, are of significant importance in the fields of materials science, pharmacy, biochemistry, and polymer science.^3−7^ They serve as an alternative to classical amphiphilic block copolymers. Their amphiphilicity and self-assembly can be fine-tuned by varying the ionic strength, temperature, and pH or by complexation with specific (bio)molecules. DHBCs with a charged block(s) are good candidates as delivery nanosystems of biomacromolecules through electrostatic complexation.^8^

Most DHBCs designed for biomedical applications typically feature a bioeliminable nonionic block, such as poly(ethylene glycol), to promote water solubility, combined with a second pH-responsive ionic block that can interact with another ionic polymer or substrate. Recently, diblock and statistical/random copolymers composed of poly[oligo(ethylene glycol) methacrylate] (POEGMA) and poly(vinyl benzyl trimethylammonium chloride) (PVBTMAC) have been successfully synthesized using a reversible addition–fragmentation chain transfer (RAFT) polymerization process.^6,7^ Studies have focused on these copolymers’ abilities to form electrostatic complexes with hydrophilic magnetic nanoparticles, MNPs, and subsequently incorporating a short DNA (a 113-base pair DNA), through dynamic and electrophoretic light scattering and cryogenic transmission electron microscopy (cryo-TEM).^6,7^ POEGMA-*b*-PVBTMAC charged DHBCs can engage in electrostatic interactions with negatively charged proteins such as insulin, while the neutral block segments (POEGMA) promote solubilization and stabilization in aqueous environments. It has been demonstrated that the electrostatic interactions between the charged block and the oppositely charged entities dictate the self-assembly and characteristics of the complexes.^6,7^ In contrast to the well-understood self-assembly of block copolymers in the liquid state, knowledge is lacking concerning their properties in the solid state.

POEGMA is a water-soluble and biocompatible polymer, highly recommended for biomedical applications, as it enhances the solubility and stabilization of the copolymer in aqueous environments.^9,10^ Additionally, the POEGMA homopolymer doped with Li salt is of great importance for energy applications. Particularly, its densely grafted macromolecular architecture, coupled with short ethylene glycol side groups, suppresses crystallinity and enhances segmental mobility, thereby engineering high ionic conductivities, that motivated studies as solid polymer electrolytes.^11−13^ Recent investigations of the molecular dynamics of POEGMA homopolymers in the dry state have unveiled the presence of a segmental process and two secondary processes, reflecting the local motions of the hydrophilic side chain into the glassy state.^14^ Furthermore, in dry amphiphilic diblock copolymers composed of POEGMA and poly(lauryl methacrylate), the strong segregation strength between the host blocks results in heterogeneous dynamics.^15^

Concerning the PVBTMAC homopolymer, it is composed of a polystyrene (PS) backbone featuring a side group bearing a strong positive charge at every repeating unit and a chloride mobile anion. The polymer has mainly been studied in aqueous solutions,^6,7,16,17^ but it has also been employed in hybrid composites that combine therapeutics, diagnostics, and sensing modalities in a single nanoparticle that is of great importance for nanomedicine.^18^

Herein, we investigate the thermodynamics, structural properties, and molecular dynamics in DHBCs composed of POEGMA and PVBTMAC blocks and the corresponding random/statistical OEGMA/VBTMAC copolymers. SAXS/WAXS measurements revealed a weak segregation strength between the parent constituents that results in dynamic homogeneity, as evidenced from TM-DSC and dielectric spectroscopy. Furthermore, a segmental relaxation of POEGMA confined between the glassy intermixed regions was revealed featuring (i) reduced *T*_g_, (ii) reduced dielectric strength, (iii) broader distribution of relaxation times, and (iv) reduced fragility compared to the POEGMA homopolymer, reflecting the structural and dynamical heterogeneities of the copolymer system.

## 2 Methods

### 2.1 Synthesis of PLMA-*b*-POEGMA

**Figure 1:**
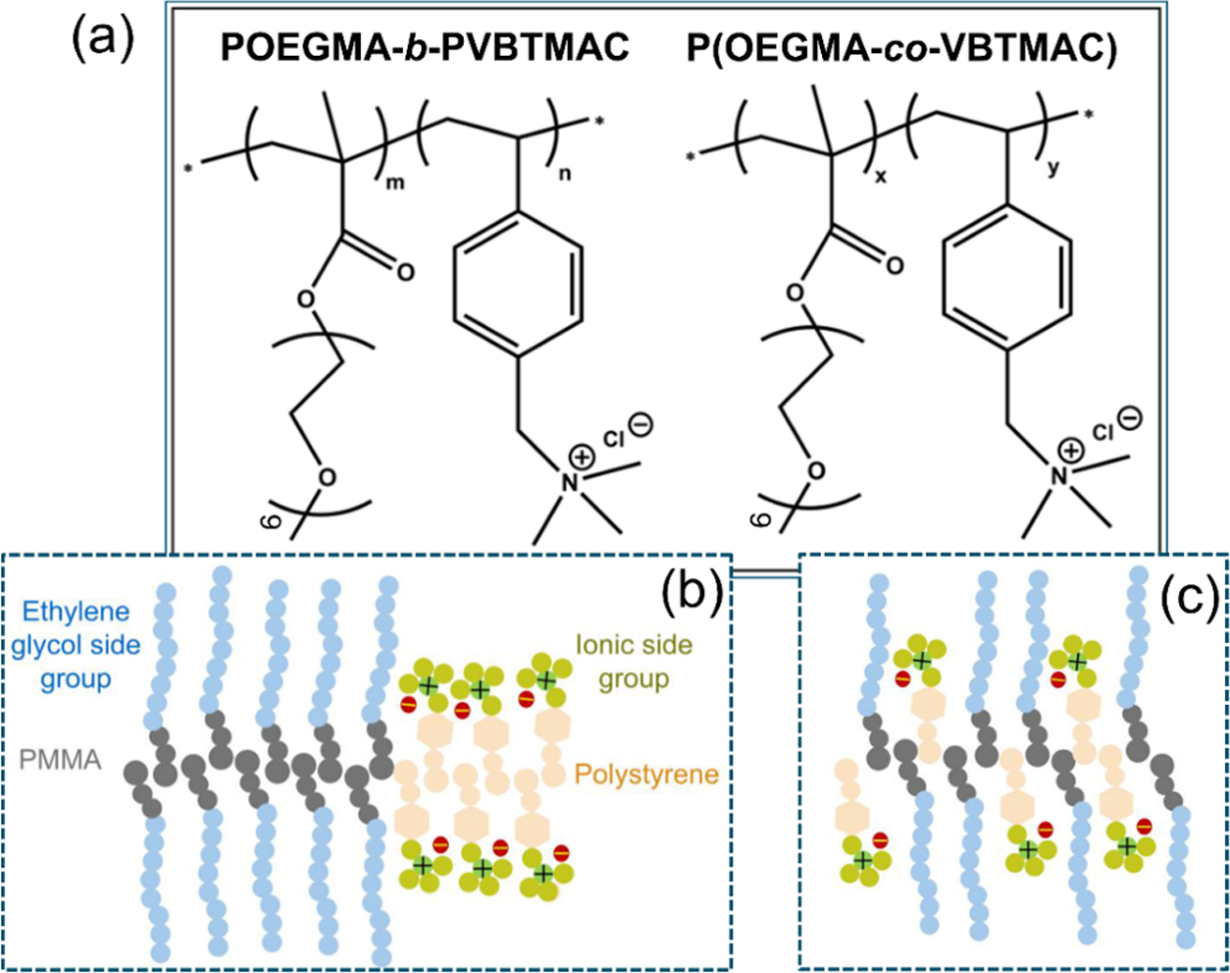
(a) Chemical structure of (a) POEGMA-*b*-PVBTMAC and P(OEGMA-*co*-VBTMAC) double hydrophilic copolymers. (b,c) Schematic representation of the block arrangement in (b) diblock and (c) random copolymers.

The synthetic procedure and molecular characterization of the POEGMA_81_-*b*-PVBTMAC_19_ and of the random copolymers are highlighted in previous studies.^6,7^ Specifically, the charged DHBCs with densely grafted macromolecular architecture were prepared by RAFT polymerization, an advantageous method for controlling the molar mass (*M*_w_) and achieving polydispersity values close to unity.^19,20^ The chemical structure of the investigated DHBCs is depicted in Figure 1, along with a schematic representation depicting specified/unspecified arrangements of the host blocks, the densely grafted macromolecular architecture, and the different side group lengths of the two blocks.

The molecular characteristics of the studied DHBCs and their respective homopolymers are listed in Table 1.

**Table 1:** Molecular Characteristics of the Studied DHBCs and Their Respective Homopolymers

sample code	*M*_n_ (g·mol^–1^)^a^	*M*_n_ (g·mol^–1^)^a^ POEGMA	PVBTMAC monomer units	POEGMA monomer units	wt %^b^ PVBTMAC
POEGMA	17,200	17,200		36	0
POEGMA_81_-*b*-PVBTMAC_19_	22,400	18,200	20	38	19
POEGMA_80_-*co*-PVBTMAC_20_	20,000		19	34	20
POEGMA_60_-*co*-PVBTMAC_40_	18,300		35	23	40
PVBTMAC	40,000		190		100

a Determined via aqueous SEC.

b Determined via ^1^H NMR in D_2_O.

### 2.2 Differential Scanning Calorimetry

Differential scanning calorimetry (DSC) measurements were performed using a Q2000 (TA Instruments) calorimeter equipped with a liquid nitrogen cooling system. Samples with a mass of approximately 5 mg were encapsulated in hermetic aluminum pans. An empty aluminum crucible was used as a reference. The weighing of the samples, pans, and lids was performed using a balance (Precisica 262SMA-FR) with a precision of 0.01 mg. Prior to the measurements, the instrument was calibrated to ensure an optimal performance. The precalibration procedure involved (i) cleaning of the cell, (ii) conditioning the cell to create an inert atmosphere using helium gas, and (iii) calibrating the LNCS baseline. Subsequently, a three-point calibration using indium (*T*_m_ = 428.8 K, Δ*H*_m_ = 28,71 J·g^–1^), mercury (*T*_m_ = 234.32 K, Δ*H*_m_ = 11.443 J·g^–1^), and Milli-Q water (*T*_m_ = 273.15 K, Δ*H*_m_ = 335 J·g^–1^) was carried out for the calibration of the enthalpy and transition temperatures. Finally, a baseline measurement was conducted with an empty cell for verifying the successful calibration of the calorimeter. Regarding the heat capacity calibration, a temperature-modulated DSC (TM-DSC) calibration was performed by employing a sapphire standard.

Additionally, TM-DSC measurements were performed in the temperature range from 143 to 473 K. In TM-DSC, a low-frequency sinusoidal perturbation is summarized to the standard DSC profile, according to *T* = *T*_0_ + β*t* + sin(ω*t*), where β is the linear cooling/heating rate, *t* is the time, *T* is the amplitude, and ω is the angular frequency.^21,22^ An amplitude of 1 K and five periods of modulation (i.e., 200, 100, 80, 60, and 40 s) were used. The respective linear heating rates, β, were extracted from the following equation
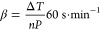
1where Δ*T* is the temperature width of the glass transition temperature, *n* is the number of modulation cycles, and *P* is the period of modulation.

### 2.3 Small/Wide-Angle X-ray Scattering (SAXS/WAXS)

SAXS and WAXS measurements were conducted using a Mat:Nordic instrument (SAXSLAB) at Chalmers Material Analysis Laboratory (CMAL), for identifying the self-assembly of POEGMA-based hydrophilic copolymers, as commonly used in the literature.^23,24^ The measurements were carried out in vacuum at room temperature. The exposure time of each sample was 15 min for both WAXS and SAXS. In SAXS, a sample–detector distance of ∼1.08 m was used, which gives a *q*-range of 0.00138–0.30717 Å^–1^. Complementarily, WAXS enables the analysis of shorter distances by employing a sample–detector distance of 0.13 m, which covers a *q*-range of 0.00381–2.2397 Å^–1^.

### 2.4 Dielectric Spectroscopy (DS)

DS measurements were carried out by using a Novocontrol Alpha frequency analyzer. The temperature protocol involved measurements from 173 to 423 K in steps of 5 K and for frequencies ranging from 10^–2^ to 10^7^ Hz, under atmospheric pressure. The dielectric cell was composed of two electrodes, each 20 mm in diameter, with the sample held at a thickness of 100 μm by Teflon spacers. Samples were prepared as melts under vacuum by pressing the electrodes to achieve the desired spacer thickness. The complex dielectric permittivity ε* = ε′ – iε″, where ε′ is the real and ε″ is the imaginary part, was obtained as a function of frequency, ω, temperature, *T*, and pressure, *P*, i.e., ε*(*T*, *P*, ω).^25,26^ The measured relaxation dynamics were fitted by the empirical equation of Havriliak and Negami (HN)
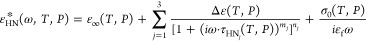
2where ε_∞_(*T*,*P*) is the high-frequency permittivity, τ_HN_(*T*,*P*) is the characteristic relaxation time in this equation, Δε(*T*,*P*) = ε_0_(*T,P*) – ε_∞_(*T,P*) is the relaxation strength, *m* and *n* (with limits of 0.2 < *m*, *mn* ≤ 1) describe the symmetrical and asymmetrical broadening, respectively, of the distribution of relaxation times, σ_0_ is the DC conductivity, and ε_f_ is the permittivity of free space. From τ_HN_, the relaxation time at maximum loss, τ_max_, is obtained analytically as follows^27^
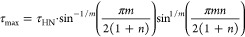
3

For analyzing the dynamic behavior, we used the imaginary part of the complex permittivity. For the interfacial/intermixed process, the derivative of the dielectric permittivity was employed for suppressing the conductivity contribution. Moreover, we employed the real part of the complex conductivity, σ′, to determine the values of the dc-conductivity.

## 3 Results and Discussion

### 3.1 Thermodynamics

The presence of possible first-order transitions in the DHBCs and their respective homopolymers can be studied with conventional DSC. The standard DSC thermograms are shown in Figure S1 upon heating. It is worth noting that the melting peak of the ethylene glycol side chains can be observed for the diblock with 80 wt % of POEGMA, reflecting the presence of bulk POEGMA nanodomains. Conversely, the melting peak is suppressed for the random copolymer with the same composition, due to its random block arrangement.

**Figure 2:**
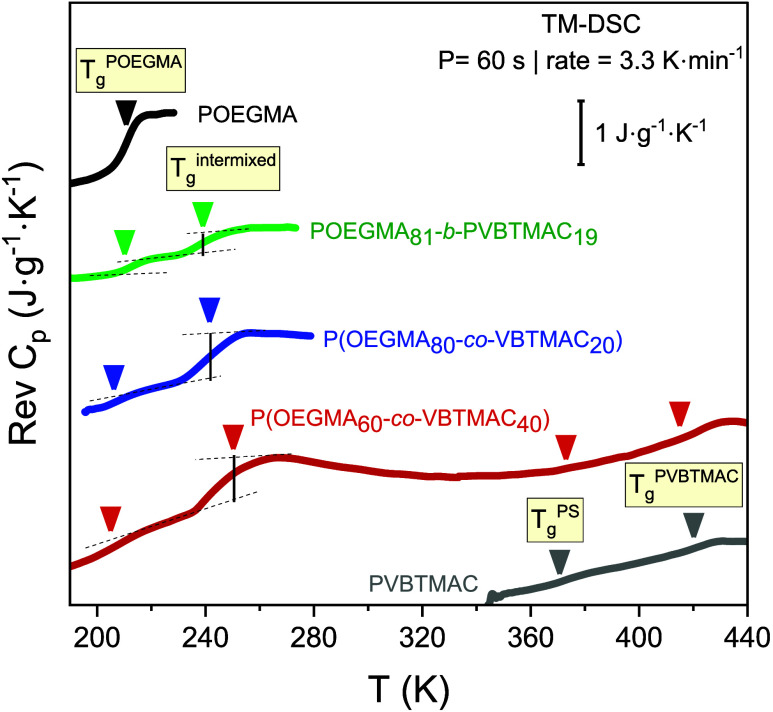
Temperature dependence of reversing heat capacity for POEGMA (black), POEGMA_81_-*b*-PVBTMAC_19_ (green), P(OEGMA_80_-*co*-VBTMAC_20_) (blue), P(OEGMA_60_-*co*-VBTMAC_40_) (red), and PVBTMAC (gray).

Insights into the glassy dynamics in DHBCs can be accurately attained through a combination of TM-DSC and dielectric measurements, where the sample is triggered by an external frequency. The TM-DSC thermograms of the investigated diblock and random DHBCs and their respective homopolymers are displayed in Figure 2.

As shown in Figure 2, the POEGMA homopolymer exhibits a *T*_g_ equal to 211 K, in accordance with the literature.^14^ On the other hand, the rigid PVBTMAC homopolymer displays two *T*_g_s at 373 and 420 K, attributed to the vitrification of the PS backbone segments (*T*_g_^PS^) and the vitrification of the PVBTMAC side chains (*T*_g_^PVBTMAC^), respectively. The ionic interactions between N^+^ and Cl^–^ ions slow down the side chain dynamics. To the best of our knowledge, it is the first time in the literature that the *T*_g_s of dry PVBTMAC are documented.

**Figure 3:**
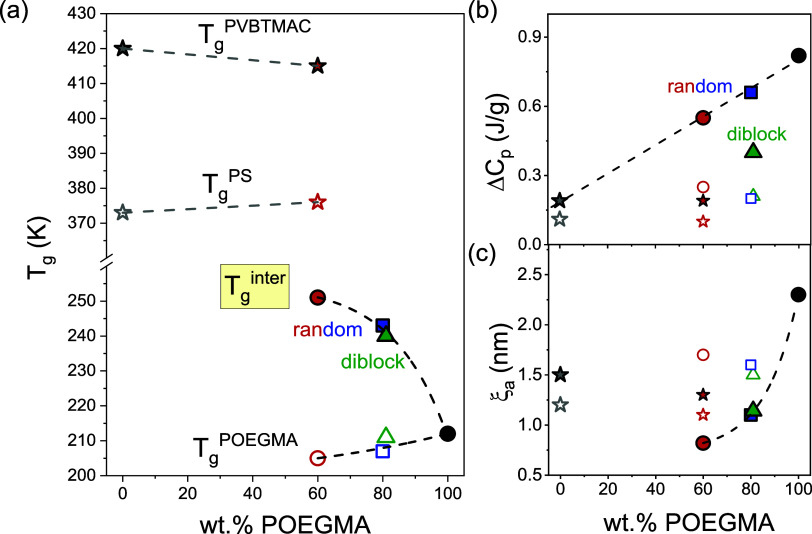
(a) Weight fraction dependence of (a) glass transition temperatures, (b) change of heat capacity, and (c) length scale of cooperativity at *T*_g_, calculated from eq 4, for POEGMA- (open symbols), intermixed- (filled symbols), PS- (stars), and PVBTMAC- (filled stars) related dynamics.

Concerning the diblock DHBC with 80 wt % of POEGMA, two *T*_g_s at 211 and 237 K, corresponding to the vitrification of bulk POEGMA (*T*_g_^POEGMA^) and intermixed POEGMA/PVBTMAC regions (*T*_g_^inter^), respectively, can be observed. The *T*_g_^inter^ is ∼31 to 46 K higher than *T*_g_^POEGMA^, reflecting the presence of intermixed regions and the hardness of the PVBTMAC blocks. By change of the block arrangement in random copolymers, the thermodynamic characteristics are similar to those of the diblock copolymer with the same composition. However, in this case, the *T*_g_^inter^ exhibits an increased change of heat capacity compared to the diblock copolymer, indicating increased degrees of freedom. With an increase in the PVBTMAC content, the intermixed *T*_g_ slightly rises due to the lower mobility of the PVBTMAC segments. Furthermore, two broad and weak *T*_g_s at 376 and 415 K can be observed, attributable to the vitrification of PVBTMAC segments, in line with the PVBTMAC homopolymer. It should be noted here that the presence of the homopolymer glass transitions in the copolymer does not necessarily imply immiscibility between the two blocks.^28^ Additionally, the *T*_g_^POEGMA^ decreases by about 2–6 K, compared to that found in the POEGMA homopolymer, indicating confinement effects, as discussed below. The values of the glass transition temperatures and the change of heat capacity are graphically presented in Figure 3(a,b) and are summarized in Table 2.

**Table 2:** Calorimetric Data Summarizing the Values of Glass Transition Temperatures (*T*_g_) and the Respective Changes of Heat Capacity (Δ*C*_p_), Extracted from TM-DSC Measurements with a Period of Modulation of 60 s

sample code	*T*_g_^POEGMA^ (K)	Δ*C*_p_^POEGMA^ (J·g^–1^·K^–1^)	*T*_g_^inter^ (K)	Δ*C*_p_^inter^ (J·g^–1^·K^–1^)	*T*_g_^PVBTMAC^ (K)	Δ*C*_p_^PVBTMAC^ (J·g^–1^·K^–1^)
POEGMA	211	0.90				
POEGMA_81_-*b-*PVBTMAC_19_	211	0.21	240	0.40		
POEGMA_80_-*co-*PVBTMAC_20_	207	0.20	243	0.66		
POEGMA_60_-*co-*PVBTMAC_40_	205	0.25	251	0.55	376	0.10
					415	0.19
PVBTMAC					373	0.11
					420	0.21

As shown in Figure 3b, in random copolymers, the change of heat capacity corresponding to the intermixed *T*_g_ decreases by decreasing the OEGMA content. Additional information about the interfacial *T*_g_ in DHBCs and how it compares with the *T*_g_ of the host homopolymers can be gained by calculating their length scales. This length scale corresponds to the size of cooperatively rearranging regions (CRRs) as initially defined by Adam and Gibbs.^29^ By employing the Donth model, the length scale of *T*_g_ can be calculated from the following equation^30^
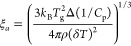
4where *k*_B_ is the Boltzmann constant, Δ(1/*C*_p_) = 1/*C*_p_^glass^ – 1/*C*_p_^liquid^, calculated at *T*_g_ and δ*T* = Δ*T*/2.5, is the mean temperature fluctuation of CRRs, and ρ is the mass density. The length scales, ξ_a_ of correlated molecules close to the observed *T*_g_s are provided in Table S1 (Figure S2) and graphically presented in Figure 3c, as a function of the POEGMA content. Particularly, for the intermixed *T*_g_, the length scale of cooperativity decreases exponentially by decreasing the POEGMA content (in the measured concentration range: 60–100 wt % POEGMA), implying distinctly smaller cooperativity regions compared to the POEGMA and PVBTMAC constituting homopolymers.

Overall, the calorimetric results indicate miscibility between the host constituents and the presence of small bulk domains primarily dependent on the copolymer composition. At this point, X-ray scattering measurements are necessary to investigate the segregation strength between the two blocks.

### 3.2 Morphology

**Figure 4:**
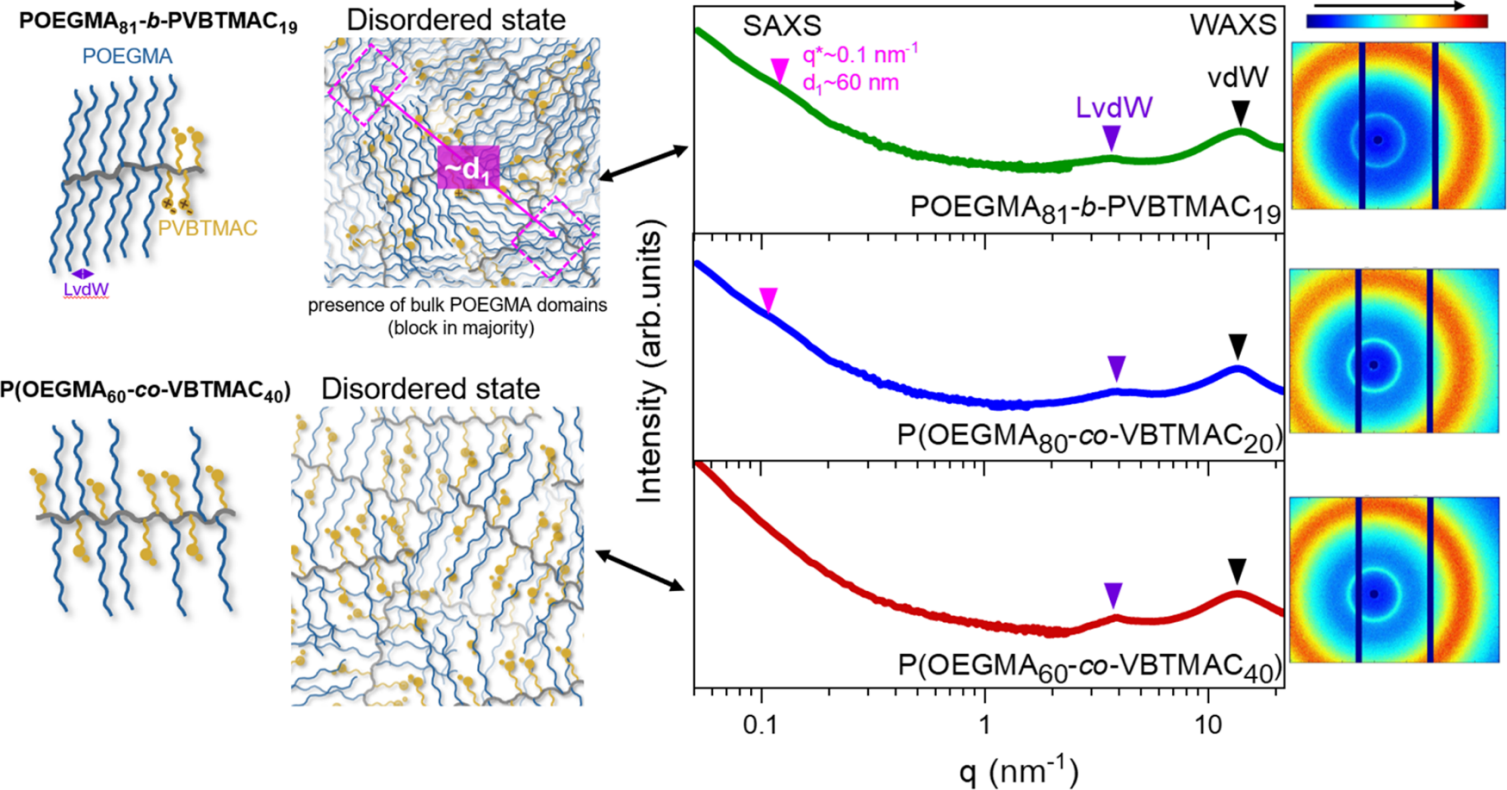
(Middle) Small-angle and wide-angle X-ray scattering (SAXS and WAXS) combined spectra of POEGMA_81_-*b*-PVBTMAC_19_ (green), P(OEGMA_80_-*co*-VBTMAC_20_) (blue), and P(OEGMA_60_-*co*-VBTMAC_40_) (red) at 298 K. (Right) 2D-WAXS images and (left) schematic representations of the microstructure in POEGMA_81_-*b*-PVBTMAC_19_ and P(OEGMA_60_-*co*-VBTMAC_40_), constructed on the basis of the SAXS/WAXS results. Specifically, on the left side, the block arrangements in (top) POEGMA_81_-*b*-PVBTMAC_19_ and (bottom) P(OEGMA_60_-*co*-VBTMAC_40_) are provided. The schematic structural illustrations present the disordered state and the inherent miscibility between the two blocks, governing the studied DHBCs. The magenta arrow indicates a typical distance between bulk POEGMA nanodomains, reflecting the presence of both intermixed and pure POEGMA regions. On the other hand, the schematic representation of P(OEGMA_60_-*co*-VBTMAC_40_) shows that the two components are homogeneously distributed and intermixed over the whole sample, i.e., there are no nanodomains due to an excess of one of the two components/monomeric units.

The self-assembly in the DHBCs can be investigated by SAXS/WAXS measurements. Figure 4 provides SAXS/WAXS data for the copolymers along with schematic representations of their microstructure at ambient temperature.

We can observe two maxima in the WAXS patterns. From the peak maximum, a correlation distance can be calculated by employing *d* = 2π*z*/*q*, where *z* (=1.23) takes into account exclusively the nearest neighbor correlations.^31^ The peak at high *q*, with an equivalent Bragg spacing of about 0.5 nm, corresponds to the van der Waals (vdW) contacts of the atoms and is known as the vdW peak. The peak at low *q*, commonly called the low van der Waals (LvdW) peak, reflects mainly the average distance between adjacent backbones and longer range intermolecular correlations.

In the SAXS data, there is a hint of a weak and broad shoulder at approximately 0.1 nm^–1^ (*d*_1_ = 2π/q* ∼ 60 nm), indicating a typical domain spacing between bulk POEGMA nanodomains, when POEGMA is the block in majority, as schematically depicted in Figure 4. This shoulder in the SAXS data reflects the difference in electron density between the intermixed and pure POEGMA regions, and it is evident both in diblock and random copolymers with 80 wt % POEGMA. In the random copolymer with 60 wt % POEGMA, the almost equimolar composition results in the absence of any weak peak or shoulder in that q-range, yielding a homogeneously dispersed intermixed/disordered state, as schematically shown in the corresponding structural illustration of Figure 4. Based on our experimental results, we believe that different compositions of both diblock and random double hydrophilic copolymers, bearing the same molar mass, would also remain in a disordered state, as the interaction parameters between the two blocks remain unchanged. Similarly, we expect that PVBTMAC-rich copolymers would also exhibit weak segregation between the two blocks and remain in a disordered state, although with a higher *T*_g_^inter^ due to the presence of more rigid segments within the intermixed domains. For enhancing the segregation strength between the two blocks, there are two possible approaches: (i) increasing the copolymer’s molar mass or (ii) doping with ions (*e.g*., Li salt) particularly for studies focused on solid polymer electrolytes. The second approach seems more promising. To sum up, the studied DHBCs are in a disordered state, reflecting the inherent miscibility between the parent homopolymers.

### 3.3 Relaxation Dynamics

To gain further insights into the compatibility between the hydrophilic blocks and elucidate how the disordered state affects the molecular dynamics, isothermal dielectric measurements were performed. The relaxation dynamics of the POEGMA homopolymer are well studied in the literature.^14,15^ On the other hand, the relaxation dynamics of the PVBTMAC homopolymer are studied here for the first time. The glassy dynamics of PVBTMAC can be discussed with respect to Figure S3, showing dielectric relaxation data of PVBTMAC in comparison with that found for polystyrene. Notably, the PVBTMAC homopolymer exhibits two relaxation processes, which we attribute to side chain and segmental backbone (i.e., polystyrene) dynamics. The former seems to exhibit large-scale segmental motions coupled to the segmental dynamics of the backbone, but below the temperature of the higher *T*_g_ (i.e., 420 K), the large-scale motions of the side chains seem to be replaced by more local side chain dynamics, which decouples from the segmental backbone dynamics. Thus, at the higher calorimetric *T*_g_^PVBTMAC^, the temperature dependence of the relaxation time changes, reflecting the unfreezing of segmental side chain dynamics with increasing temperature. The relaxation dynamics of the PVBTMAC homopolymer is shown in Figure S3(b,c) of the Supporting Information.

**Figure 5:**
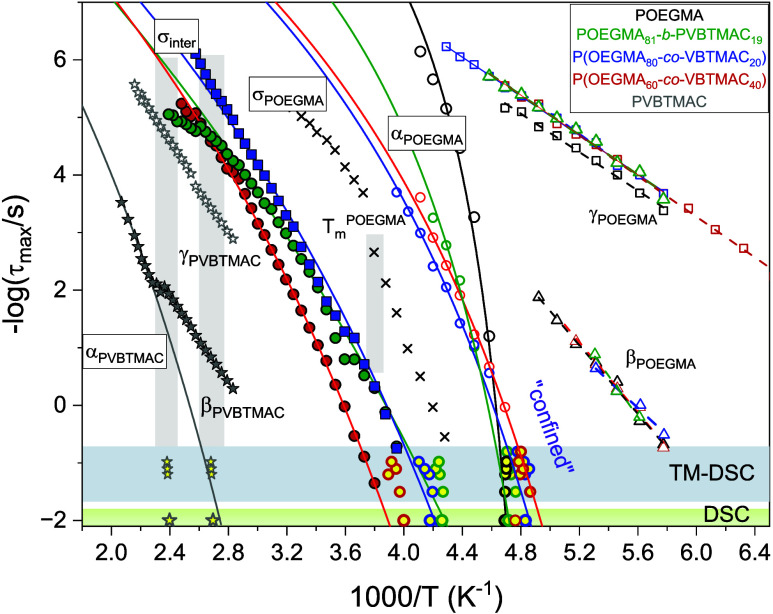
Relaxation times as a function of reciprocal temperature, depicting the local γ_POEGMA_ (open squares), β_POEGMA_ (open triangles), α_POEGMA_ (open circles), σ_POEGMA_ (crossed symbols), σ_inter_ (filled circles), γ_PVBTMAC_ (open stars), and α_PVBTMAC_ - β_PVBTMAC_ (filled stars) processes, for the POEGMA (black), POEGMA_81_-*b*-PVBTMAC_19_ (green), P(OEGMA_80_-*co*-VBTMAC_20_) (blue), P(OEGMA_60_-*co*-VBTMAC_40_) (red), and PVBTMAC (gray), upon heating. The yellow symbols come from TM-DSC measurements. The solid and dashed lines indicate fits by the VFT (eq 6) and Arrhenius (eq 5) equation, respectively.

A relaxation map depicting all the dielectrically active processes is shown in Figure 5, and the observed processes are termed as γ_POEGMA_, β_POEGMA_, α_POEGMA_, σ_POEGMA_, σ_inter_, α_PVBTMAC_, β_PVBTMAC_, and γ_PVBTMAC_. The figure includes DSC and TM-DSC data depicting the calorimetric glass transitions. The relaxation times were obtained by curve fitting of the dielectric data shown in Figures S4–S6.

Starting from low temperatures, the DHBCs exhibit the γ_POEGMA_ process featuring a distribution of relaxation times reminiscent with that found in the POEGMA homopolymer, as depicted in Figure S4. It exhibits an Arrhenius temperature dependence according to
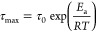
5where τ_0_ is the relaxation time in the limit of an infinitely high temperature and *E*_α_ is the activation energy. As listed in Table 3, the activation energies of the γ_POEGMA_ and β_POEGMA_ processes are reminiscent with that of the POEGMA homopolymer and they are unaffected by the POEGMA content, indicating the same molecular origin.^14^

**Table 3:** Summary of VFT Parameters, Dielectric Glass Transition Temperature, and Fragility for the Investigated DHBCs and Their Respective Homopolymers

sample code	–log(τ_0_^#^/s)^a^	*B* (K)	*T*_0_ (K)	*T*_g_ (K)^b^	m*
	*α*_POEGMA_ process				
POEGMA	12	580 ± 40	195 ± 1	214 ± 1	149
POEGMA_81_-*b*-PVBTMAC_19_	12	1310 ± 50	171 ± 2	211 ± 2	75
POEGMA_80_-*co*-PVBTMAC_20_	12	2170 ± 80	138 ± 3	206 ± 3	42
POEGMA_60_-*co*-PVBTMAC_40_	12	2100 ± 200	137 ± 5	203 ± 5	42
	***σ***_**inter**_**process**				
POEGMA_81_-*b*-PVBTMAC_19_	12	4700 ± 100	88 ± 5	233 ± 5	22
POEGMA_80_-*co*-PVBTMAC_20_	12	3870 ± 70	119 ± 3	238 ± 3	29
POEGMA_60_-*co*-PVBTMAC_40_	12	4200 ± 30	127 ± 1	257 ± 1	28
	*α*_PVBTMAC_ process				
PVBTMAC (***α***_**PVBTMAC**_)	12	5800 ± 300	184 ± 10	365 ± 2	27

a Held fixed.

b Dielectric *T*_g_ at τ = 100 s.

By increasing the temperature, the α_POEGMA_ and the σ_inter_ processes can be observed in the dielectric spectra and their relaxation times follow the Vogel–Fulcher–Tammann (VFT) dependence as
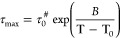
6where τ_0_^*#*^ is the relaxation time in the limit of an infinitely high temperature, *B* is the activation parameter, and *T*_o_ is the “ideal” glass transition temperature located below the conventional *T*_g_, dielectrically defined at 100 s. The VFT and Arrhenius parameters of the dielectrically active processes along with the values of the observed glass transitions are provided in Tables 3 and S2, respectively.

#### 3.3.1 Intermixed Process

The σ_inter_ process is evident exclusively in the derivative of the dielectric permittivity, and it is the most pronounced and well-defined process in the experimental frequency window. As depicted in Figures 5 and S5, it is located between the segmental dynamics of the host homopolymers and freezes at the calorimetric *T*_g_^inter^, implying homogeneous dynamics into intermixed POEGMA/PVBTMAC domains. The latter verifies the weak segregation strength evidenced in SAXS/WAXS measurements and the strong compatibility between the two blocks. Additionally, this process coincides with the crossing frequency of the real and imaginary parts, suggesting that it mainly reflects long-range charge mobility. However, this ion dynamics freezes at the *T*_g_^inter^, reflecting a coupling between the ionic motions and segmental intermixed dynamics. Therefore, this relaxation process has both ionic and structural characteristics. These points are detailed below in connection with the dc-conductivity results. It is worth noting that the σ_inter_ process is unaffected by the block arrangement (i.e., sequential, random copolymer), reflecting its molecular origin. Moreover, the presence of intermixed domains is verified by the dielectric strength and shape parameters of the interfacial processes, as depicted in Figure S7. Specifically, their dielectric strengths are almost equal to the summation of those found in the host constituents (see Figure S7a). Additionally, the low-frequency shape parameter of the interfacial processes attains values in between those found in the parent homopolymers. By decreasing the POEGMA content in random DHBCs, the intermixed segmental process slows down, and the associated *T*_g_^inter^ increases due to an increasing amount of the “harder” and less mobile (i.e., glassy) PVBTMAC segments. Overall, the weak segregation strength between the two blocks and their inherent miscibility result in intermixed POEGMA/PVBTMAC domains and thus *dynamic homogeneity*.

#### 3.3.2 “Confined” POEGMA Dynamics

As it is well-documented in the literature even for fully miscible blends or for mixtures of small molecules, there are additional dielectric processes at temperatures below the *T*_g_^inter^, suggesting the presence of dynamic heterogeneities.^32−34^ Specifically, at *T* < *T*_g_^inter^, the α_POEGMA_ process is evident in the dielectric spectra, as depicted in Figure S6. This process exhibits (i) reduced dielectric strength, (ii) broader distribution of relaxation times, (iii) reduced *T*_g_, and (iv) decreased fragility (see below) compared to the POEGMA homopolymer. Therefore, this process is attributed to the segmental relaxation in POEGMA nanodomains, restricted by the confinement between the glassy intermixed regions.^32−39^ The effect of confinement becomes more pronounced with an increase in the PVBTMAC content and by variation of the sequence of the covalently bonded polymer blocks (e.g., from diblock to random), as schematically depicted in Figure S8. Furthermore, the “confined” α_POEGMA_ process in DHBCs shows a distinctly different temperature dependence (i.e., fragility) as compared to that of bulk POEGMA.

**Figure 6:**
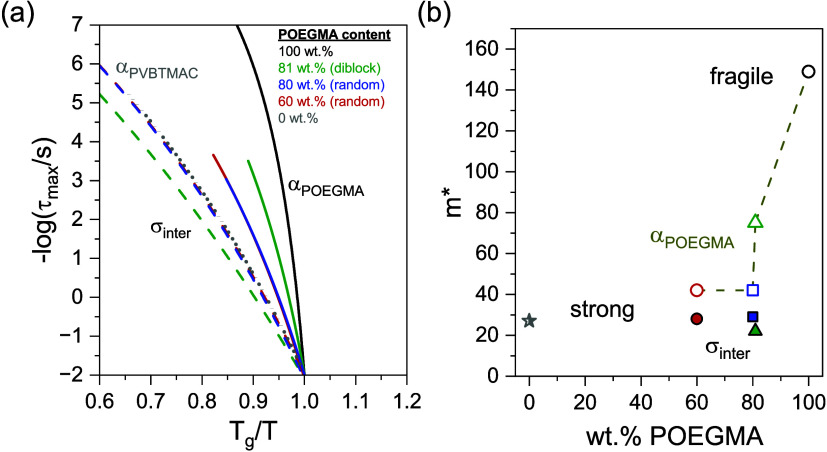
(a) Relaxation times vs normalized temperature depicting the POEGMA (solid lines), PVBTMAC (gray dotted line), and intermixed (dashed lines) segmental processes for the studied DHBCs. (b) Extracted values of fragility or steepness index as a function of POEGMA composition for the α_POEGMA_ (open symbols), α_PVBTMAC_ (semifilled star), and σ_inter_ (filled symbols).

To quantify the temperature dependence of the α_POEGMA_ and σ_*inter*_ processes close to the associated glass transitions, the calculation of steepness index (fragility), *m*, is illustrative. The steepness index can be calculated as m* = *BT*_g_/[2.303(*T*_g_ – *T*_0_)^2^] or can be extracted from the slope at *T*_g_ in the fragility plot (see Figure 6a).^40^ The extracted values are given in Table 3 and Figure 6b. The σ_*inter*_ relaxation process in DHBCs exhibits distinctly reduced fragility compared to that found for the segmental relaxation of POEGMA and similar to that found for the PVBTMAC homopolymer, suggesting that the hard segments of the latter polymer have a major influence on the nature of this intermixed relaxation. The α_POEGMA_ process of the diblock copolymer displays a 2-fold decrease in fragility compared to the homopolymer. An additional 2-fold decrease can be observed following a random arrangement of the blocks. Therefore, the DHBCs can be characterized as “strong” glass-forming polymers.

The results from the present study can be compared with the segmental dynamics in model bottlebrush polymers consisting of a poly(2-bromoisobutyryloxyethyl methacrylate) (PBiBEM) backbone and grafted *n*-butyl acrylate (PBA) chains and with amphiphilic diblock copolymers based on PLMA-*b*-POEGMA.^15,41^ In the latter system, the amphiphilic nature of copolymers induces nanophase segregation and gives rise to heterogeneous dynamics. On the other hand, in model bottlebrush polymers based on PBiBEM, the grafted macromolecular architecture was found to impart *dynamic homogeneity* to the backbone and side chain dynamics, as a result of the weak segregation strength. A similar situation was found in the current study of DHBCs. Therefore, the inherent miscibility between the POEGMA and PVBTMAC blocks results in disordered copolymers with *homogeneous dynamics* along with confined POEGMA segmental motions.

### 3.4 Conductivity

**Figure 7:**
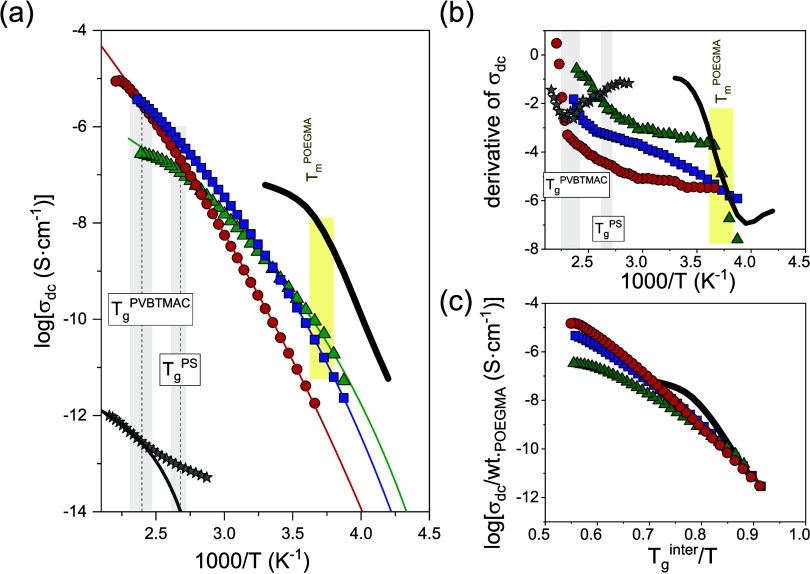
(a) Temperature dependence of the dc-conductivity for POEGMA (black line), POEGMA_81_-*b*-PVBTMAC_19_ (green up-triangles), POEGMA_80_-*co*-PVBTMAC_20_ (blue squares), POEGMA_60_-*co*-PVBTMAC_40_ (red circles), and PVBTMAC (gray stars). (b) Derivative of the dc-conductivity as a function of the reciprocal temperature. The gray- and yellow-colored areas indicate the glass transition temperatures of PVBTMAC and the melting of POEGMA side chains, respectively. (c) Conductivity normalized by the weight fraction of the POEGMA block as a function of *T*_g_^inter^/*T*.

The dc-conductivity is extracted from the plateau of the real part of the complex conductivity, as illustrated in Figure S9. The temperature dependencies of the extracted values of dc-conductivities are plotted as a function of reciprocal temperature in Figure 7a.

At ambient temperature, the dc-conductivity of the copolymers is about 10^–9^ S/cm, which is about 2 orders of magnitude lower than that found for the POEGMA homopolymer. This reflects the intermixing between the host constituents and thus the coupling between σ_inter_ and the dc-conductivity, as evident from the Walden plot of Figure S10. As depicted in Figure 7b, the ionic conductivity of the diblock copolymer is affected by the melting of POEGMA side chains, reflecting the presence of bulk POEGMA nanodomains and verifying the calorimetric results. Additionally, the derivative of the dc-conductivity reflects the glass transition temperatures of PVBTMAC in the investigated copolymers. After normalization with the weight fraction of POEGMA and the intermixed *T*_g_ (see Figure 7c), the conductivities almost coincide into a single curve at lower temperatures, implying that the glass transition temperature of intermixed domains dictates the dc-conductivity. This further suggests a coupling between the ionic dynamics and intermixed segmental dynamics.

## 4 Conclusions

In this work, we studied the molecular dynamics, thermodynamics, and self-assembly in DHBCs based on the POEGMA and PVBTMAC parent homopolymers. Both diblock and random copolymers were examined. SAXS results revealed a weak segregation strength between the host blocks, resulting in intermixed POEGMA/PVBTMAC domains. As a result, the homogeneous dynamics governs the dielectric spectra. The intermixed process freezes at a temperature, *T*_g_^inter^, between the glass transition temperatures of the POEGMA and PVBTMAC homopolymers. It should also be mentioned that the glass transition temperatures of the dry PVBTMAC homopolymer, attributed to the backbone (i.e., polystyrene) and side chain segmental dynamics, are documented for the first time in the literature. The dc-conductivity is primarily dictated by *T*_g_^inter^, verifying the homogeneous structural nature of DHBCs, and the coupling between the ionic motions and the segmental dynamics in the intermixed regions. At temperatures below *T*_g_^inter^, the segmental relaxation of confined POEGMA segments can be detected, featuring (i) reduced *T*_g_, (ii) reduced dielectric strength, (iii) broader distribution of relaxation times, and (iv) reduced fragility compared to the POEGMA homopolymer. Overall, this study shows that excellent mixing of the two homopolymers can be achieved and that it is possible to customize the desired properties of copolymers by mixing suitable homopolymers in appropriate concentrations.

## Supporting Information Available

Additional standard DSC, TM-DSC, and dielectric data (PDF)
