# Supplementary material for: Molecular Dynamics and Self-Assembly in Double Hydrophilic Block and Random Copolymers
Source: J Phys Chem B. 2024 Nov 5;128(45):11267–76. doi: 10.1021/acs.jpcb.4c05398 (PMC11571219; doi:10.1021/acs.jpcb.4c05398)
Supplement: Supplementary file 1 — jp4c05398_si_001.pdf [file jp4c05398_si_001.pdf]

Supporting Information

Molecular Dynamics and Self-assembly in Double Hydrophilic Block and Random Copolymers

Achilleas Pipertzis,[1] Angeliki Chroni,[2] Stergios Pispas,[2] Jan Swenson[1]*

[1]*Department of Physics, Chalmers University of Technology, 41296, Gothenburg, Sweden*

[2]*Theoretical and Physical Chemistry Institute, National Hellenic Research Foundation, 48 Vassileos Constantinou Ave., 11635 Athens.*

Correspondence: Achilleas Pipertzis
Email: achilleas.pipertzis@chalmers.se

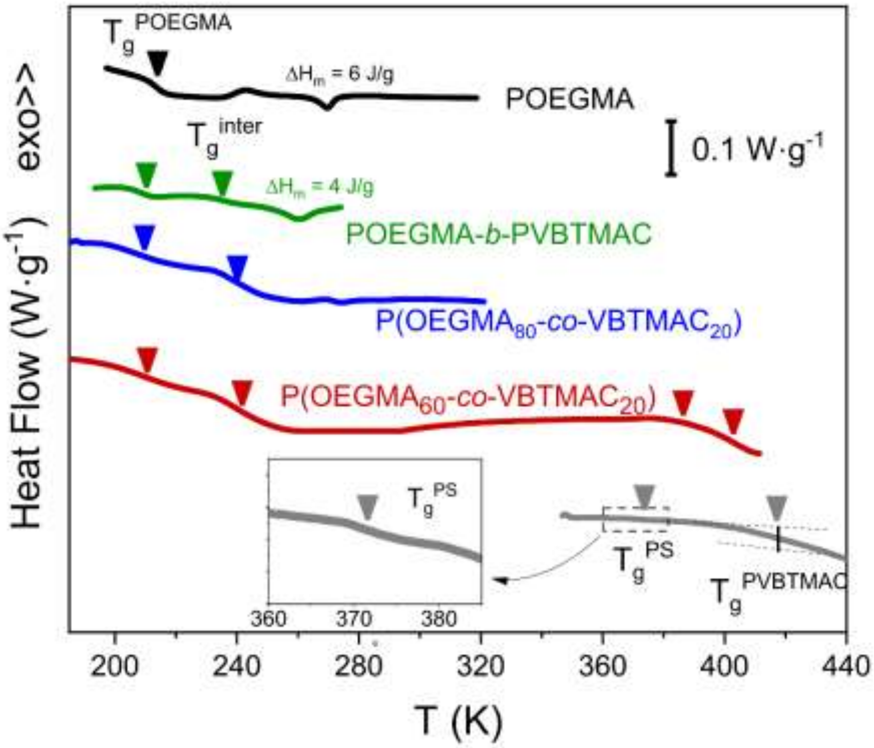

Figure S1. Standard DSC thermograms for POEGMA (black), $POEGMA_{81}$-*b*-$PVBTMAC_{19}$ (green), P($OEGMA_{80}$-*co*-$VBTMAC_{20}$) (blue), P($OEGMA_{60}$-*co*-$VBTMAC_{40}$) (red) and PVBTMAC (gray), upon heating with a rate of 3.3 K·min^{-1}.

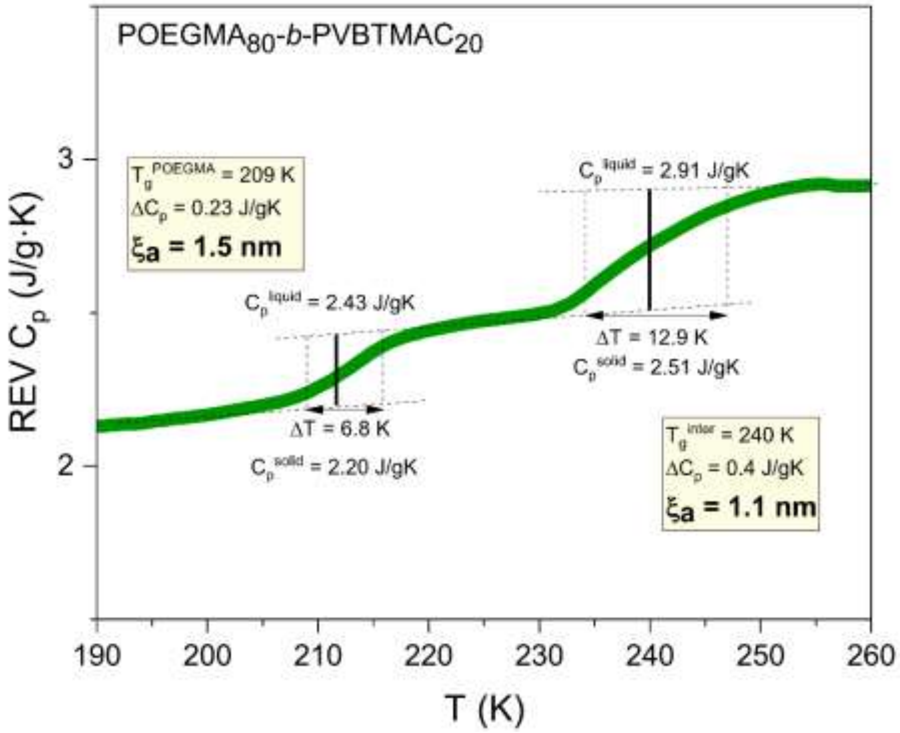

Figure S2. TM-DSC thermogram for the diblock copolymer. The parameters used in the Donth model are presented.

Table S1. Length Scale of Cooperativity Close to the Observed T_gs in DHBCs and their Corresponding Homopolymers.

Sample code	ξ_a^{POEGMA} (nm)	ξ_a^{inter} (nm)	$\xi_a^{PVBTMAC}$ (nm)
POEGMA	2.3		
POEGMA$_{81}$-*b*-PVBTMAC$_{19}$	1.5	1.1	
POEGMA$_{80}$-*co*-PVBTMAC$_{20}$	1.6	1.1	
POEGMA$_{60}$-*co*-PVBTMAC$_{40}$	1.7	0.8	1.3[a]<br>1.1[b]
PVBTMAC			1.5[a]<br>1.2[b]

[a]associated with T_g^{PS}, [b]associated with $T_g^{PVBTMAC}$

The dielectric loss curve of PVBTMAC is compared and contrasted with that found for polystyrene with different molar masses in Figure S3.

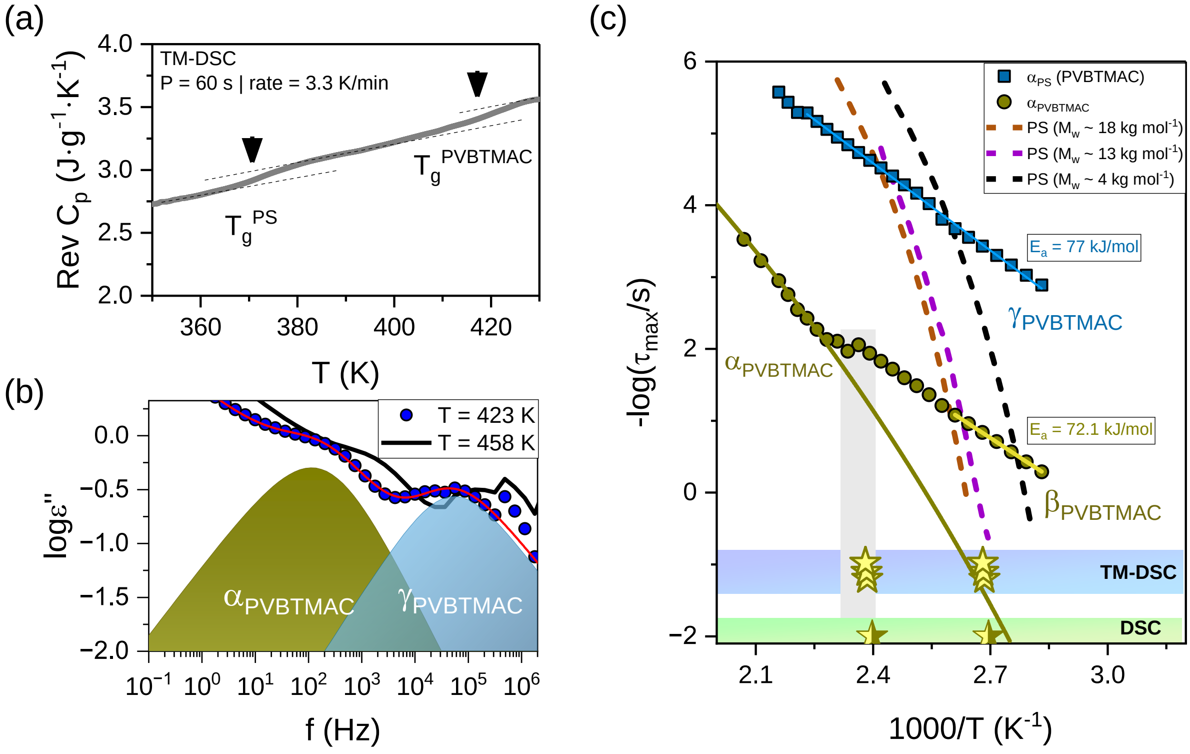

Figure S3. (a) TM-DSC thermogram for the PVBTMAC homopolymer. The vertical arrows indicate the two glass transitions. (b) Frequency dependence of dielectric spectra for PVBTMAC (black lines and blue symbols at two temperatures: $T = 423$ K (blue symbols) and $T = 458$ K (black line). (c) Relaxation map depicting $\gamma_{PVBTMAC}$ (cyan squares) and $\alpha_{PVBTMAC}$ /$\beta_{PVBTMAC}$ (dark yellow circles) for PVBTMAC. The dashed lines represent literature data for

polystyrene with different molar masses; 4 kg·mol^{-1} (black), 13 kg·mol^{-1} (purple) and 18 kg·mol^{-1} (orange).[1] The dark-yellow and cyan solid lines represent fits to VFT and Arrhenius equation, respectively.

The PVBTMAC homopolymer exhibits two secondary processes, termed as, β_{PVBTMAC}, and $\gamma_{\mathrm{PVBTMAC}}$, at temperatures below the T_g^{PS} and T_g^{PVBTMAC}, respectively. The $\gamma_{\mathrm{PVBTMAC}}$ process is about 2 orders of magnitude faster compared to the β_{PVBTMAC}. These processes display relatively weak temperature dependences, following the Arrhenius equation with activation energies of approximately 70 kJ·mol^{-1}. Therefore, they reflect local motions associated with the PS backbone and side chain motions in the glassy state.

At temperatures above the T_g^{PVBTMAC}, the segmental motions unfreeze and contribute to a stronger VFT temperature dependence. These segmental motions freeze at the T_g^{PS} (at τ = 100 s), reflecting the segmental backbone (*i.e.* polystyrene) dynamics. However, there is a change from VFT to Arrhenius temperature dependence at T_g^{PVBTMAC}, reflecting the unfreezing of the side chain dynamics.

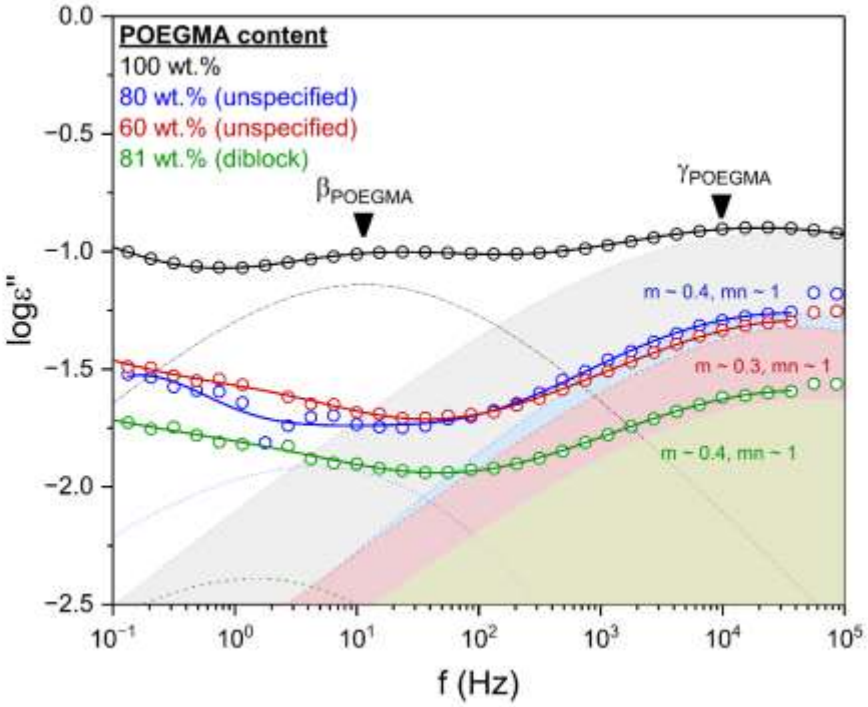

Figure S4. Dielectric loss curves of POEGMA (black symbols), P(OEGMA$_{80}$-*co*-VBTMAC$_{20}$) (blue symbols), P(OEGMA$_{60}$-*co*-VBTMAC$_{40}$) (red symbols) and POEGMA$_{81}$-*b*-PVBTMAC$_{19}$ (green symbols). The colored areas represent the γ_{POEGMA} process.

Table S2. Arrhenius Parameters for the Investigated Copolymers and their Corresponding Homopolymers.

Sample code	$-\log(\tau_0/s)$	E_a (kJ·mol^{-1})	$-\log(\tau_0/s)$	E_a (kJ·mol^{-1})
	***β* process**		***γ* process**	
POEGMA	16.5 ± 0.6	57.02	13.1 ± 0.3	31.94
$POEGMA_{81}$-*b*-$PVBTMAC_{19}$	19 ± 1	69.1	13.7 ± 0.3	33.1
$POEGMA_{80}$-*co*-$PVBTMAC_{20}$	14 ± 1	58.3	13.5 ± 0.2	32.4
$POEGMA_{60}$-*co*-$PVBTMAC_{40}$	17 ± 1	58.3	13.9 ± 0.3	33.8

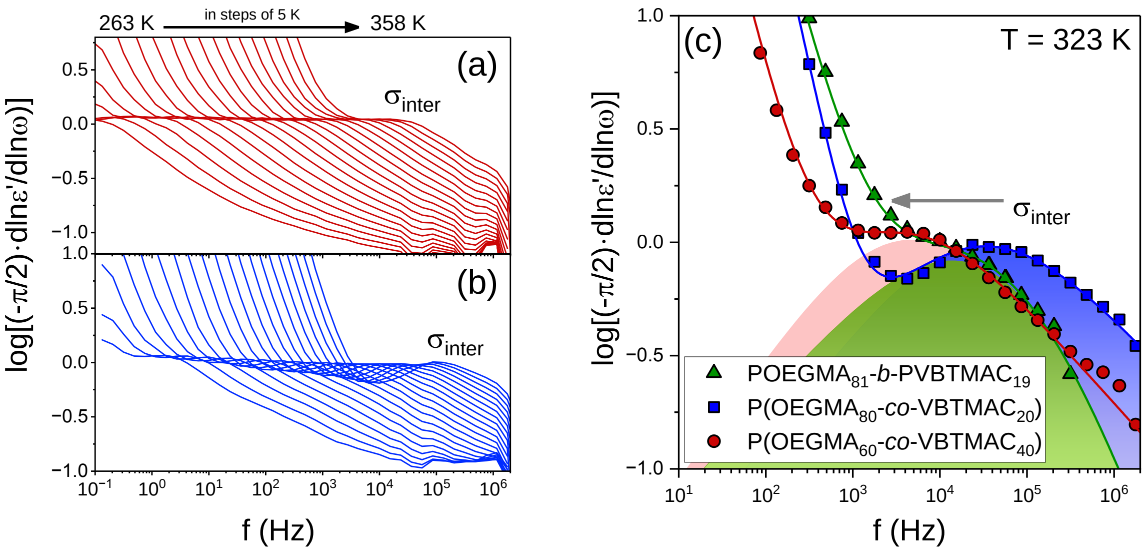

Figure S5. (a) Derivative of dielectric permittivity spectra for P($OEGMA_{80}$-co-$VBTMAC_{20}$) from 263 K to 358 K in steps of 5 K. (b) Comparison of the derivative of the dielectric permittivity for $POEGMA_{81}$-*b*-$PVBTMAC_{19}$ (green up-triangles), P($OEGMA_{80}$-*co*-$VBTMAC_{20}$) (blue squares), P($OEGMA_{60}$-*co*-$VBTMAC_{40}$) (red circles), at a fixed temperature of 323 K. The colored areas indicate the σ_{inter} process. The grey arrow shows the slowing-down of the σ_{inter} process.

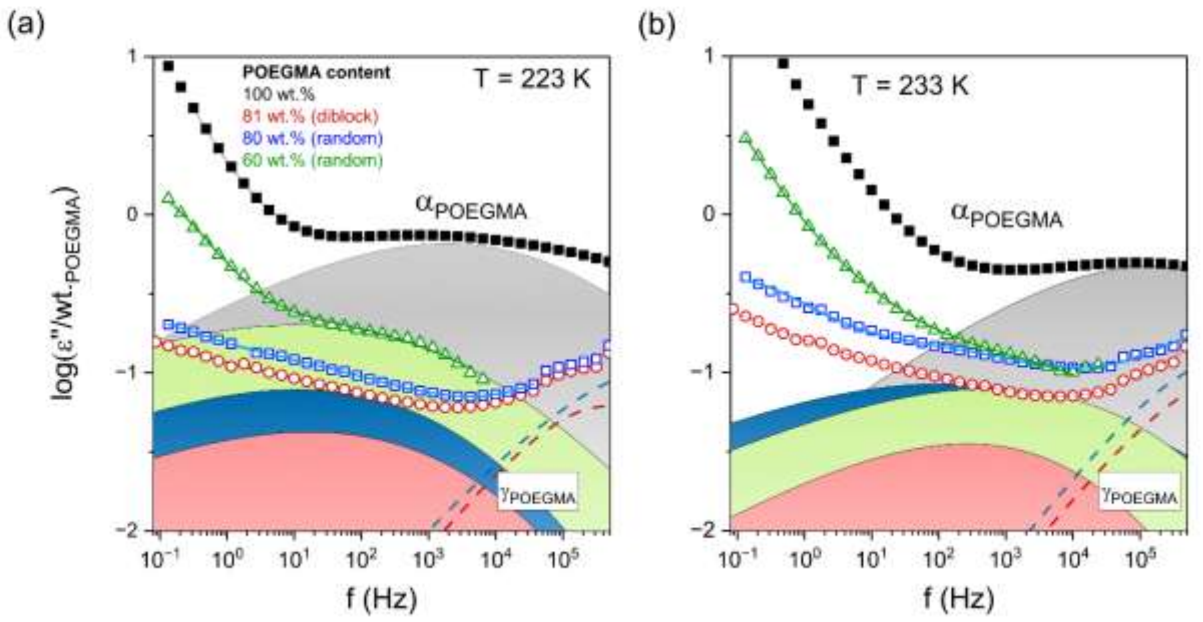

Figure S6. Dielectric loss curves normalized to the weight fraction of POEGMA for POEGMA (black squares), P(OEGMA$_{80}$-*co*-PVBTMAC$_{20}$) (blue symbols), P(OEGMA$_{60}$-*co*-PVBTMAC$_{40}$) (red symbols) and POEGMA$_{81}$-*b*-PVBTMAC$_{19}$ (green symbols). The shadowed areas and dashed lines represent simulations to the α_{POEGMA} and γ_{POEGMA} process, respectively.

The dielectric strength and the shape parameters for the different segmental relaxation processes are provided in **Figure S7**.

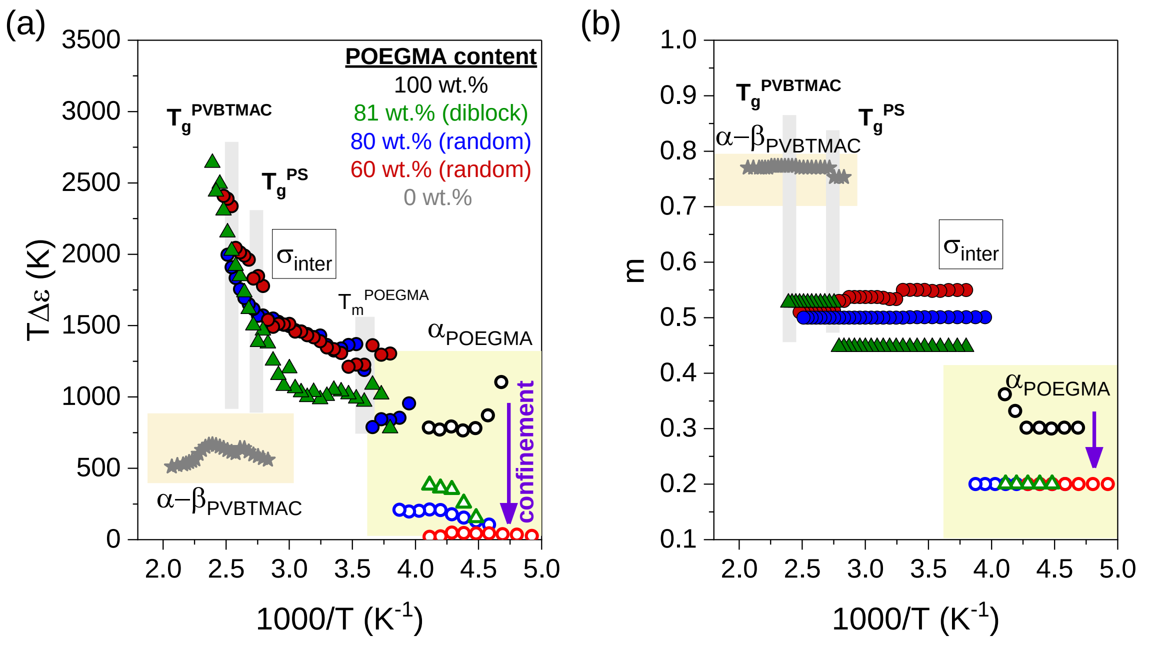

Figure S7. (a) Inverse temperature dependence of (a) the dielectric strength and (b) the low-frequency shape parameter of the α_{inter} and α_{POEGMA} relaxations for the unspecified DHBCs with 80 wt.% (blue symbols) and 60 wt.% (red symbols) of POEGMA and the diblock DHBC with 81 wt.% of POEGMA (green symbols). The data for the segmental relaxations of the host homopolymers are also included.

The σ_{inter} in DHBCs displays a dielectric strength equal to the summation of the dielectric strengths of the homopolymers' segmental dynamics. Additionally, the low-frequency shape parameter exhibits values in between those found in the segmental processes of the host homopolymers. Therefore, the σ_{inter} reflects ion motions coupled to segmental dynamics of intermixed POEGMA/PVBTMAC domains.

Figure S8 schematically shows the confined POEGMA nanodomains within the glassy intermixed regions.

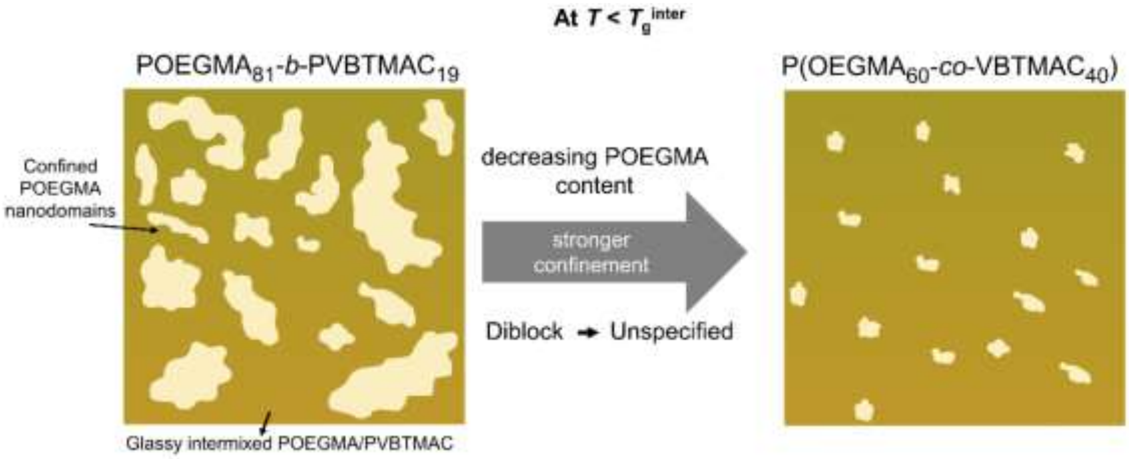

Figure S8. Schematic representation depicting the confined POEGMA domains into the glassy intermixed regions for POEGMA$_{81}$-*b*-PVBTMAC$_{19}$ (left) and P(OEGMA$_{60}$-*co*-VBTMAC$_{40}$) (right), at $T < T_g^{inter}$.

The values of dc-conductivity were extracted from the plateau of the real part of complex conductivity., as illustrated in **Figure S9**.

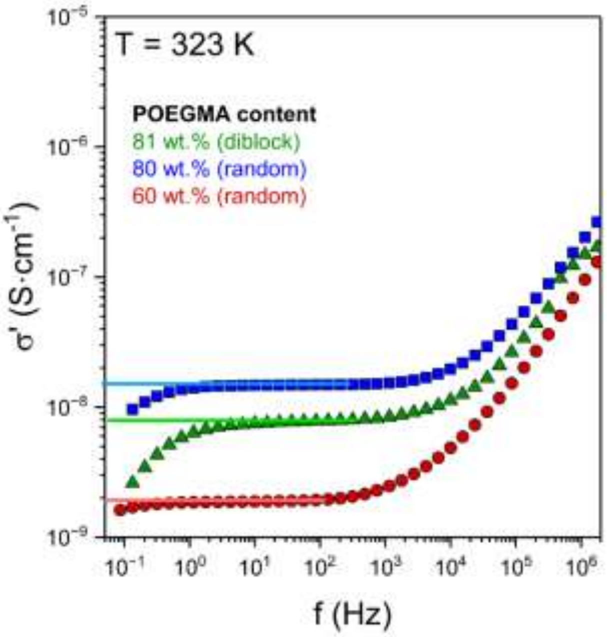

Figure S9. Frequency dependence of the real part of the complex conductivity for the unspecified DHBCs with 80 wt.% (blue symbols) and 60 wt.% (red symbols) of POEGMA and the diblock DHBC with 81 wt.% of POEGMA (green symbols) at T = 323 K.

The dc-conductivity are plotted as a function of the σ_{inter} relaxation times in **Figure S10**.

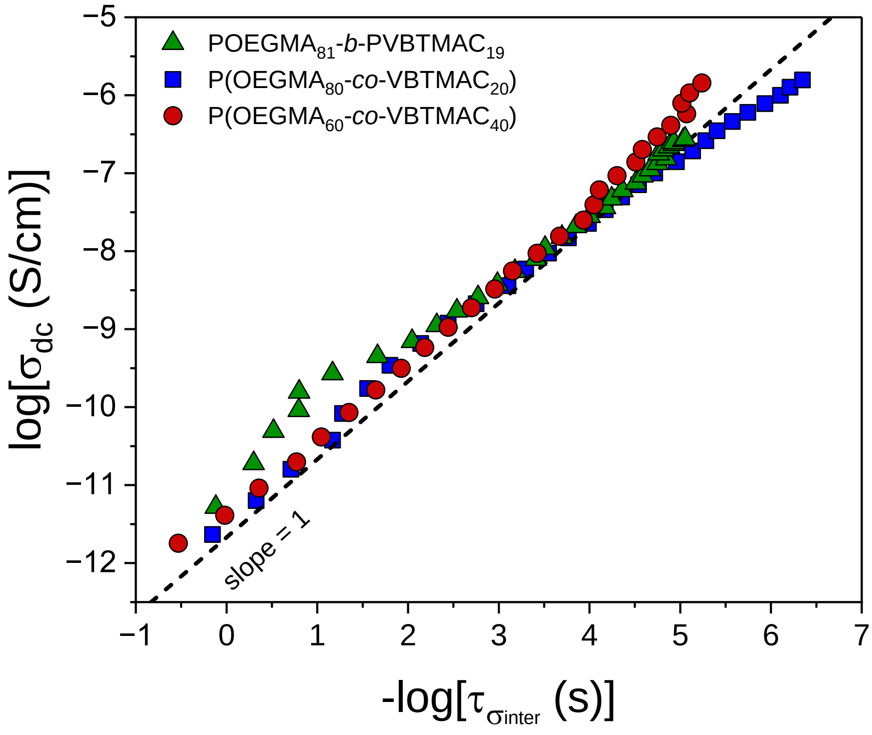

Figure S10. Dc-conductivity as a function of the σ_{inter} relaxation times in a log-log representation (i.e. Walden plot).

REFERENCES

(1) Pipertzis, A.; Hossain, M. D.; Monteiro, M. J.; Floudas, G. Segmental dynamics in multicyclic polystyrenes. *Macromolecules* **2018**, *51* (4), 1488-1497.
